# The emotional states and associated behavioral responses (flexible‐adaptive behaviors vs. inflexible‐maladaptive behaviors) of cancer patients during the SARS‐CoV‐2 outbreak: A multi‐center cross‐sectional study in Italy

**DOI:** 10.1002/cam4.7442

**Published:** 2024-07-01

**Authors:** Giuseppe Deledda, Sara Poli, Matteo Giansante, Eleonora Geccherle, Giovanna Fantoni, Incoronata Romaniello, Farina Gabriella, Matteo Verzè, Fabrizio Nicolis, Stefania Gori

**Affiliations:** ^1^ Clinical Psychology Unit IRCCS Sacro Cuore Don Calabria Hospital Verona Italy; ^2^ O.S.S. Trinità Borgomenero Italy; ^3^ Department of Oncology ASST Fatebenefratelli Sacco Milan Italy; ^4^ Medical Direction IRCCS Sacro Cuore Don Calabria Hospital Verona Italy; ^5^ Oncology Department IRCCS Sacro Cuore Don Calabria Hospital Verona Italy

**Keywords:** cancer patients, COVID‐19 outbreak, maladaptive behavior, psychological reactions

## Abstract

**Objective:**

Distress during SARS‐CoV‐2 outbreak affected also cancer patients' well‐being. Aim of this study was to investigate patient' reactions and behavior (flexible‐adaptive vs. inflexible‐maladaptive) during the SARS‐CoV‐2 outbreak.

**Methods:**

A cross‐sectional survey was designed with a self‐report questionnaire, “the ImpACT questionnaire,” developed for the study. Regression analysis was performed on data.

**Results:**

Four hundred and forty five cancer patients from 17 Italian regions participated in the study. 79.8% of participants were female (mean age of 58 years). 92.6% of participants reported feeling vulnerable to COVID‐19 contagion; 75.6% reported helpless, 62.7% sad, 60.4% anxious, and 52.0% anger. Avoidance of thinking about coronavirus is the principal maladaptive behavior that emerged. Participants who reported feeling anxious were more likely to have fear of staff being infected with COVID‐19 (OR = 3.01; 95% CI = 1.49–6.30) and to have disrupted sleep due to worry (OR = 2.42; 95% CI = 1.23–4.83). Younger participants reported more anxiety (OR = 0.97; 95% CI = 0.94–1.00); men reported feeling calm more than women (OR = 2.60; 95% CI = 1.27–5.43).

**Conclusions:**

Majority of cancer patients reported serious concerns regarding SARS‐CoV‐2 infection; reliable information and psychological support must be offers to respond to these needs.

## 1 BACKGROUND

A considerable amount of evidence has shown the correlation between the SARS‐CoV‐2 outbreak and the increased risk of cancer patients suffering adverse psychological effects,^1^, ^2^, ^3^, ^4^ including worsening of anxiety,^5^ distress,^6^, ^7^, ^8^ depression,^9^ loss of energy, and quality of life.^10^, ^11^

Overexposure to SARS‐CoV‐2 related information has also increased distress and the inability to disengage from information flow.^6^, ^12^

As SARS‐CoV‐2 has become a major stressor, several studies reported increased stress levels in nurses, physicians, and radiographers.^13^, ^14^ The organizational characteristics of the workplace, along with the social appreciation of the job position, significantly influence overall satisfaction with life.^15^

Nonetheless, cancer patients generally have been of good opinion regarding the medical attention they received during the pandemic, and satisfaction with received care is a good predictor of emotional patients wellbeing.^3^, ^6^

Other studies have focused on specific aspects of psychological health during the SARS‐CoV‐2 outbreak, seeking to identify which aspects might best explain protective and destructive emotional responses in patients.^3^, ^16^, ^17^ Specifically, several studies have shown that dispositional psychological flexibility is key in ensuring good psychological health.^18^

Psychological flexibility has been shown to be highly conducive in the establishment and maintenance of mental wellbeing^19^, ^20^ and enables individuals to adapt to changing circumstances by altering their behavior without compromising their personal values.^18^ Psychological flexibility is defined as “one's intentions to display situation‐appropriate behaviors”^21^ and has been shown to be a robust predictor of effective problem‐focused and emotion‐focused coping strategies during stressful events.^22^

Psychological flexibility is a core component of ACT and may be crucial in understanding how individuals are affected by, and cope with, the significant challenges brought on by cancer and its treatments. Psychological flexibility is defined as the ability to identify and adapt to situational demands in an attempt to improve longer‐term outcomes in a way that is personally meaningful. It has been associated with improved psychological health, quality of life, and well‐being in both clinical and non‐clinical populations including both distress‐related and positive outcomes (e.g., benefit finding) in cancer survivors. Furthermore, psychological flexibility has been shown to be amenable to change over time, presenting a potential target for interventions.^23^, ^24^

In contrast, psychologically inflexible behaviors, which are dysfunctional and contextually non‐adaptive, manifests as rigid psychological reactions.^25^ For instance, inflexible behaviors can include the radical avoidance of interpersonal relationships, the excessive consumption of food, or an increased use of alcohol and/or cigarettes.^3^ In such a context, people can feel overwhelmed by uncontrollable fear. In contrast, embracing (rather than avoiding) inner discomfort and accepting unwanted thoughts, while also engaging in values‐based action, increases resilience during adversity.^26^, ^27^

Our study aimed to investigate the emotional states and associated behavioral responses (flexible‐adaptive behaviors vs. inflexible‐maladaptive behaviors) of cancer patients during the SARS‐CoV‐2 outbreak in Italy.

## 2 METHODS

### 2.1 Procedure

We have developed a cross‐sectional study with a snowball sampling strategy that focused on recruiting oncological patients from Italian oncology operating units was utilized.

Italian patients over 18 years of age with oncological conditions, not affected by COVID‐19, were eligible to participate in the study.

Potential respondents were invited to complete an anonymous survey, and, after informed consent was obtained, a digital or paper version (depending on access to the hospital for medical visits) of the questionnaire was administered.

Ethics approval was obtained from the Ethical Committee for Clinical Trials of the Provinces of Verona and Rovigo in Northern Italy (Prog. 2642 CESC), which conformed to the principles embodied in the Declaration of Helsinki.

Data collection took place over 1 month after the WHO had declared the SARS‐CoV‐2 outbreak (April 2020–May 2020). The data that support the findings of this study are available from the corresponding author upon reasonable request.

### 2.2 Measures

#### 2.2.1 Outcome variable

In order to evaluate emotional states and their associated behavior, we developed a 31‐item “ImpACT” self‐report questionnaire. The result of self‐rated emotional states was the primary outcome variable for the study. Three different emotional states, occurring during the pandemic (April 2020–May 2020), were assessed by asking participants whether or not they agreed with the following statements: “I feel anxious,” “I feel angry,” and “I feel calm.”

In the first part of the questionnaire, study participants were asked to respond to the question, “How do you feel about the coronavirus right now?” by reporting their emotional state (calm, indifferent, sad, feeling at fault towards family members, feeling powerless, angry, nervous or restless, worried, anxious, scared, or panicked) as compared to that of prior to the SARS‐CoV‐2 outbreak.

#### 2.2.2 Primary explanatory variable

Participant behavior was the primary explanatory variable for the study. The second part of the questionnaire was developed in order to elaborate on the behaviors associated with the emotional states identified in part one. The “ImpACT” questionnaire has been used previously for patients with various conditions.^3^ It was specifically adapted for cancer patients by changing the words “doctor” to “oncologist” and “medical center” to “oncological center.”

Respondents used a 5‐point Likert scale, ranging from 0 “never true” to 5 “always true”, to rate different behaviors with respect to both the internal context (thoughts, emotions, and physical sensations) and the external context (external stimuli, including the context of care).

Consistent with the ACT model (based on functional contextualistic science),^19^, ^20^ two types of behaviors were investigated: (1) adaptive and flexible behaviors (e.g., openness to emotional experience and the acceptance of aversive emotions, thoughts, or physical sensations); and (2) inflexible/maladaptive behaviors (e.g., experiential avoidance function, including the avoidance or suppression of unwanted emotions with alcohol or other substances or by using distraction techniques) in an attempt to control and redirect internal experience.

#### 2.2.3 Effect modifiers variables

The sociodemographic variables potentially influencing medical and psychological aspects were controlled for in a multivariable analysis. This allowed us to isolate the effects of sociodemographic, medical, and psychological factors that may affect the self‐rating of emotional states.

Sociodemographic variables included gender, age, education history, residential location, marital status, employment status, economic losses related to the SARS‐CoV‐2 outbreak, parental status, family network, household size and layout (number of rooms, garden, balcony, etc.), cohabitation status before and during quarantine, and social isolation regulations. Respondents were also asked for information regarding their life context, including their principal sources of information regarding SARS‐CoV‐2 and COVID‐19 symptom management. Participants were also asked about their trust in said sources and any role the information may have played in providing psychological support.

Medical variables included the participants' specific pathology, physical health status, medical history, and treatment phase (diagnostic, active therapy, follow‐up, palliative care).

Psychological variables included psychological health status, psychiatric/psychological history, emotional status as related to SARS‐CoV‐2 and COVID‐19, behaviors in response to the fear of developing COVID‐19 (including avoidance behaviors and the use of substances such as alcohol).

### 2.3 Statistical analysis

Summary statistics were calculated for all variables. The 31 items of the ImpACT questionnaire were condensed from a 5‐point Likert scale into a binary response (“Never true,” “Rarely to always true”). Items 5, 17, and 31 were excluded from further analyses due to a lack of variation in the responses given. Participant sex, age, and the ImpACT questionnaire item responses were analyzed using univariable logistic regression models to explore their association with three different dependent variables describing patients' emotional states during the pandemic: anxiety, anger, and calmness. Emotions were assessed via agreement (yes or no) with the statement: “I feel anxious,” “I feel angry,” and “I feel calm.” Only variables signifiantly associated (*p*‐value < 0.2) with the three different emotional states were included in the full logistic regression model. The Akaike Information Criterion (AIC) was used to compare candidate models. Goodness‐of‐fit measures were estimated for these models. All *p*‐values were two‐tailed, with a significance level of 0.05. Analyses were performed using R software, version 3.6.1.

## 3 RESULTS

### 3.1 Participants

The sample consisted of 445 cancer patients from across 17 Italian regions, with most participants coming from three regions of northern Italy (Veneto, Lombardy, Piedmont). The sociodemographic characteristics of participants are shown in Table 1. 79.8% of participants were female, while the mean age was 58 years. 66.3% of participants held a high school certificate or higher education degree. 69.9% were married or in a relationship, and 76.6% reported having children, of which 49.2% had more than two. The year of cancer diagnosis ranged from 1987 to 2020, and 25.3% of participants reported having had a relapse. The most common oncological diagnosis was found to be breast cancer (36.2%). 59.6% of patients reported being in the active phase of treatment, and 33.18% in the follow‐up phase. The most commonly reported cancer therapy was chemotherapy (55.1%). 24.2% of participants reported having had a psychological problem before the SARS‐CoV‐2 outbreak, with anxiety and depression being the most common disorders (9.7% and 6.7% of cases, respectively).

**TABLE 1:** Participants demographics and clinical characteristics of overall sample.

Characteristic	Patients
	(*N* = 445)
Gender, *n* (%): Male	90 (20.2)
Age, median [IQR]	58 [51–68]
Marital status, *n* (%)	
Married or in a relationship	311 (69.9)
Separeted or divorced or widowed	80 (18.0)
Single	53 (11.9)
Missing	1 (0.2)
Number of children, *n* (%)	
0	103 (23.1)
1	122 (27.4)
2+	219 (49.2)
Missing	1 (0.2)
Children age, *n* (%)	
18–30	98 (22.0)
<18	58 (13.0)
>30	178 (40.0)
Missing	111 (24.9)
Education, *n* (%)	
High school	206 (46.3)
Degree or post degree	89 (20.0)
Primary or middle school	150 (33.7)
Work condition, *n* (%)	
Employed	180 (40.4)
Retired	175 (39.3)
Housewife	49 (11.0)
Unemployed	33 (7.4)
Student	2 (0.4)
Missing	6 (1.3)
With whom did you live before the lockdown (before 9 March 2020)? *n* (%)	
Spouse or partner	170 (38.2)
Spouse or partner and children	133 (29.9)
Alone	64 (14.4)
Extended family	33 (7.4)
Children	23 (5.2)
Parents	14 (3.1)
Missing	8 (1.8)
With whom do you live after the lockdown (9 March to 3 May 2020)? *n* (%)	
Spouse or partner	165 (37.1)
Spouse or partner and children	129 (29.0)
Alone	58 (13.0)
Extended family	33 (7.4)
Children	30 (6.7)
Parents	15 (3.4)
Missing	15 (3.4)
Home, *n* (%)	
With a garden	195 (43.8)
With balcony or terrace	100 (22.5)
Large house	69 (15.5)
Three‐room apartment	52 (11.7)
One or two‐room apartment	22 (4.9)
Missing	7 (1.6)
Cancer, *n* (%)	
Breast	161 (36.2)
Gastrointestinal	64 (14.4)
Urogenital	30 (6.7)
Pelvic	21 (4.7)
NET	10 (2.2)
Pulmonary/Thoracic	10 (2.2)
Head and neck	4 (0.9)
Melanoma	4 (0.9)
Lymphoma	3 (0.7)
Other	35 (7.9)
Missing	103 (23.1)
Time since diagnosis, years, median [IQR]	2 [1–6]
Treatment phase, *n* (%)	
Active therapy	265 (59.6)
Follow up	147 (33.1)
Diagnostic	5 (1.1)
Palliative care	4 (0.9)
Missing	24 (5.4)
Type of treatment, *n* (%)^a^	
Chemotherapy	147 (33.0)
Hormone therapy	41 (9.2)
None	19 (4.3)
Radiotherapy	9 (2.0)
Other	48 (10.8)
Missing	189 (42.5)
Recurrence, *n* (%)	
No	301 (67.6)
Yes	102 (22.9)
Missing	42 (9.4)
Psychological issues, *n* (%)	
No	332 (74.6)
Yes	106 (23.8)
Missing	7 (1.6)
Types of pre‐existing psychological issues, *n* (%)	
Anxiety	43 (9.7)
Depression	30 (6.7)
Anxiety or depression	26 (5.8)
Other	8 (1.8)
Missing	338 (76.0)
Time since diagnosis of psychological issues, years, median [IQR]	12 [5–20]

Abbreviations: IQR, interquartile range; NET, neuro endocrine tumors.

^a^ Patients may report more than one treatment; therefore, percentages do not sum to 100%.

Most participants (75.1%) reported that they considered the Ministry of Health, Civil Protection and Italian National Institute of Health to be trusted sources of information. Newspapers and television were trusted by around half of participants (54.6%), whereas medical professionals such as oncologists (27.2%), physicians (21.3%), and nurses (11.2%) were trusted by fewer participants. Mass media (17.1%), relatives (13.9%), psychologists (4.9%), social media (3.6%), and radio broadcasts (0.2%) were trusted to varying degrees.

Most participants reported being satisfied with the information received from oncologists (85.9%), nurses (81.0%), and psychologists (72.1%), whereas the responses given by physicians (66.3%) and emergency room staff (58.3%) proved to be appreciated by a lower proportion of participants. Further to this, although 87.0% of individuals reported keeping themselves informed about SARS‐CoV‐2 and to feel confused about the information they had received. Most patients (97.6%) reported strictly adhering to the physical distancing and PPE regulations that were stipulated by the government and medical professionals. Just over half of participants (59.8%) reported feeling safe in their oncology unit. 56.5% feared to some degree that medical staff would fall ill with COVID‐19, and 51.3% said they were afraid of disruption to ongoing medical care. 33.9% of participants reported having contacted their oncologist more frequently than so in the past, and 12.8% reported having canceled appointments with the oncologist. Most participants (74.5%) reported that the relationship with their partner and their openness towards their relatives had not changed by that stage of the pandemic, and 58.1% declared to have remained in contact with other cancer sufferers. 21.6% of participants said they did not leave home for fear of being exposed to SARS‐CoV‐2. 36.3% of participants preferred to not leave the house, and 38.0% avoided all contact with others. Most participants (87.7%) reported significant changes to their lives as compared to before the SARS‐CoV‐2 outbreak. 56.2% reported feeling more alone than in the past, and 34.5% reported being unsatisfied with life.

### 3.2 Regression analysis

In the multivariable model, after controlling for potential confounders, three different clusters of emotional states were considered (a) feeling calm, (b) feeling anxious, and (c) feeling angry, and their association with the items of the ImpACT questionnaire and sociodemographic variables were investigated.

#### 3.2.1 Cluster of participants who feel “calm”

About half of participants (45.5%) reported feeling calm during the investigated period of the COVID‐19 pandemic, with most participants reporting not feeling indifferent to the situation (93.5%).

Participants who reported feeling calm showed less fear of the future (OR = 0.20; 95% CI = 0.09–0.43; *p*‐value < 0.001), suffered less insomnia due to worry (OR = 0.46; 95% CI = 0.25–0.83; *p*‐value = 0.011), experienced fewer suicidal thoughts (OR = 0.10; 95% CI = 0.00–0.63; *p*‐value = 0.041), and were less likely to turn to religious faith for comfort (OR = 0.47; 95% CI = 0.24–0.91; *p*‐value = 0.025). More men reported feeling calm than women (OR = 2.60; 95% CI = 1.27–5.43; *p*‐value = 0.010) (Table 2).

**TABLE 2:** Multivariate logistic regressions. Dependent variables identify participants who reported feeling calm.

Predictors	I feel calm		
	Odds Ratios	CI	*p*
Gender: Male	2.60	1.27–5.43	**0.010**
Q15 **I am afraid of the future** Rarely to always true	0.20	0.09–0.43	**<0.001**
Q20 **I cannot sleep because I am worried about coronavirus** Rarely to always true	0.46	0.25–0.83	**0.011**
Q25 **I am worried about economic losses** Rarely to always true	0.58	0.29–1.16	0.125
Q26 **I have had suicidal thoughts** Rarely to always true	0.10	0.00–0.63	**0.041**
Q30 **I think faith is helping me** Rarely to always true	0.47	0.24–0.91	**0.025**
Hosmer‐Lemeshow goodness of fit *χ* ^2^(8), *p* value	3.98		0.859
Observations	257		
*R* ^2^ Tjur	0.237		

*Note*: Dependent variables identify participants who reported feeling calm.

Abbreviation: CI, confidence interval.

#### 3.2.2 Cluster of participants who feel “anxious”

Participants who reported feeling anxious were more likely to be afraid that medical staff would become unwell with COVID‐19 (OR = 3.01; 95% CI = 1.49–6.30; *p*‐value = 0.003). They also reported a lack of sleep due to worry (OR = 2.42; 95% CI = 1.23–4.83; *p*‐value = 0.011), using medication to manage anxiety (OR = 4.04; 95% CI = 1.43–13.34; *p*‐value = 0.013), and relying on religious faith for comfort (OR = 3.47; 95% CI = 1.62–7.69; *p*‐value = 0.002). Younger participants tended to report being more anxious (OR = 0.97; 95% CI = 0.94–1.00) (Table 3).

**TABLE 3:** Multivariate logistic regressions. Dependent variables identify participants who report feeling anxious.

	I feel anxious		
Predictors	Odds Ratios	CI	*p*
Age	0.97	0.94–1.00	**0.043**
Q12 **I fear medical staff can get sick** Rarely to always true	3.01	1.49–6.30	**0.003**
Q16 **I enjoy the things I do** Rarely to always true	0.15	0.01–1.24	0.122
Q19 **I kept in contact with other patients** Rarely to always true	1.78	0.88–3.65	0.111
Q20 **I cannot sleep because I am worried about coronavirus** Rarely to always true	2.42	1.23–4.83	**0.011**
Q25 **I am worried about economic losses** Rarely to always true	2.11	0.92–4.94	0.081
Q27 **I asked for psychological help** Rarely to always true	2.23	0.81–6.68	0.131
Q28 **I am using more medicine to help me handle anxiety** Rarely to always true	4.04	1.43–13.34	**0.013**
Q30 **I think faith is helping me** Rarely to always true	3.47	1.62–7.69	**0.002**
Hosmer‐Lemeshow goodness of fit χ^2^(8), *p* value	8.55		0.355
Observations	204		
*R* ^2^ Tjur	0.283		

*Note*: The dependent variables identify participants who report feeling anxious.

Abbreviation: CI, confidence interval.

In addition, 81.6% of participants reported feeling unsure about their future, 47.0% feeling agitation, 60.4% feeling anxious, 6.2% feeling panicked, and 41.4% having problems sleeping due to concerns about coronavirus. Regarding the SARS‐CoV‐2 outbreak, 62.7% of participants reported feeling sad, 75.6% feeling helpless, 13.2% feeling guilty, 16.3% feeling uncertain and out of control, while 5.2% reported having had suicidal thoughts. 89.9% of participants reported feeling worried, 97.1% reported a heightened awareness of any potential COVID‐19 symptoms (e.g., a cough or breathing difficulties), 70.6% reported being afraid of being infected with SARS‐CoV‐2, and 27.3% reported their fear of losing a loved one to COVID‐19. Almost all participants (92.6%) reported feeling vulnerable with regard to developing COVID‐19. 89.1% reported trying to avoid thinking about coronavirus. 22.7% of participants reported having sought psychological help, while 16.5% reported having taken psychotropic drugs for anxiety, and a further 59.2% reported using relaxation practices to moderate stress.

#### 3.2.3 Cluster of participants who feel “angry”

Participants who reported feeling angry were more likely to feel lonely (OR = 3.58; 95% CI = 1.98–6.63; *p*‐value = 0.019) and more likely to report avoiding thinking about coronavirus (OR = 0.34; 95% CI = 0.13–0.82; *p*‐value <0.001) (Table 4).

**TABLE 4:** Multivariate logistic regressions. Dependent variables identify participants who reported feeling angry.

	I feel angry		
Predictors	Odds Ratios	CI	*p*
Q04 **I try to avoid thinking about coronavirus** Rarely to always true	0.34	0.13–0.82	**0.019**
Q21 **I feel more lonely** Rarely to always true	3.58	1.98–6.63	**<0.001**
Q25 **I am worried about economic losses** Rarely to always true	1.77	0.84–3.83	0.136
Hosmer‐Lemeshow goodness of fit χ^2^(8), *p* value	1.82		0.986
Observations	216		
*R* ^2^ Tjur	0.115		

*Note*: Dependent variables identify participants who reported feeling angry.

Abbreviation: CI, confidence interval.

Furthermore, 52.0% of participants reported experiencing anger, and 7.8% reported anger towards those who break the rules.

## 4 DISCUSSION

Our results suggest that many cancer patients have faced the SARS‐CoV‐2 outbreak with a high degree of concern for their own health and for that of their loved ones.

Most study participants reported changes in their lives since the SARS‐CoV‐2 outbreak began, with many feeling more alone than in the past. Further to this, about a third of those surveyed reported being unsatisfied in life.

Unlike other published studies, in which participants reported an excess of SARS‐CoV‐2 information,^6^, ^12^ it seems that Italian cancer patients were generally satisfied with the information they obtained from their oncologists.

Indeed, most participants reported feeling well informed about SARS‐CoV‐2 by the oncologist and very safe in their cancer care center.

Consistent with other findings, in which it emerged that during lockdown, 35.2% of patients had canceled an appointment with their specialist doctor due to their fear of becoming infected with SARS‐CoV‐2 and that 29.4% had done so after lockdown.^3^ About one in 10 of the cancer patients surveyed here had canceled appointments with their oncologists. Further to this, about a third had contacted their doctor more so than in the past.

For each of the emotional states investigated in our study, the associated behavioral reactions (adaptive and flexible behaviors vs. maladaptive and inflexible behaviors) were analyzed.

Almost all of the sample reported paying close attention to possible COVID‐19 symptoms. This concern was managed in different ways, ranging from feeling aware of the situation but calm (for almost half of the sample) to emotional states characterized by worry, nervousness, anxiety, helplessness, feelings of guilt, sadness, and/or anger (with anger being directed towards those breaking lockdown rules especially). Such changes in emotional state would manifest as disrupted sleeping patterns; however, no evidence was apparent of increased alcohol use, food intake, or smoking among participants as means of avoiding unpleasant emotions.

Male participants reported feeling calmer than women, being less fearful of the future, and suffering less insomnia due to worry.

Participants who reported feeling anxious had more concerns about the health of medical staff, experienced more sleep disruption due to worry, had a greater reliance on their faith, and a greater propensity to use psychotropic medications to manage anxiety.

Participants who reported feeling anger during the SARS‐CoV‐2 outbreak showed a greater tendency to try and avoid thinking about coronavirus.

In line with a previous study,^3^ some behaviors arguably served an experiential avoidance function (e.g., asking for reassurance may reflect an attempt to avoid or control fear (item 10), and increasing food intake or alcohol use be done so in an effort to avoid negative emotions (item 22)). Experiential avoidance appears to be a highly dysfunctional strategy in contexts such as pandemics and is an important vulnerability factor for poor psychological health^17^, ^25^, ^26^, ^27^, ^28^ that correlates with increased anxiety, distress, depression,^3^ and insomnia.^29^, ^30^

Furthermore, angrier participants were at greater risk of feeling lonely and reported avoiding thinking about coronavirus more often. Generally speaking, such thought suppression tends to have a paradoxical effect and actually increases rumination of thoughts, resulting in increased discomfort, anxiety, frustration and anger.

Importantly, studies have shown that feelings of loneliness often precipitate psychological problems. Pre‐existing depression was identified in 12% of participants, while another study has demonstrated that individuals experiencing loneliness during the pandemic were 4.5 times at higher risk of depression.^31^ This correlates with other research suggesting that loneliness is an unintended consequence of COVID‐19 restrictions and that it aggravates pre‐existing depression.^32^

Finally, one in five of the cancer patients that participated in our study sought support from a psychologist to help manage their fears and alleviate emotional suffering.

### 4.1 Strengths and limitations

The main difficulty in this study has been distinguishing between emotional states due to the SARS‐CoV‐2 outbreak and those potentially arising as a consequence of oncological pathology. The questionnaire was carefully designed with this in mind. Subjects were contacted and recruited directly by the researchers, who collected the data primarily using paper questionnaires. As a direct approach, this may have encouraged the participation of a greater number of individuals than an open advertisement requesting participants would have done; however, the “snowball sampling strategy,” as a recruiting method, may have created a possible selection bias. Furthermore, data cannot be generalized due to the nature of a questionnaire built ad hoc to analyze cancer patients' perspectives in this particular situation.

### 4.2 Clinical implications

Assessing cancer patient's behavior and reaction during a huge stressful event such as COVID‐19 outbreak could be extremely important from a clinical point of view. If not recognized and treated adequately, these psychological effects could have strong impacts on cancer patients' psychophysical health, quality of life, and social dynamics (both at home and at work). This could potentially translate into an increased burden on the national healthcare system.

Therefore, the way in which healthcare professionals recognize and respond to patients' needs, facilitate understanding of information, and how they offer appropriate and timely emotional support is ever more relevant.

## 5 CONCLUSIONS

These results suggest that the majority of cancer patients participating in our study had concerns about developing COVID‐19 and about their health in general. Furthermore, feelings of uncertainty regarding the management and implementation of their cancer treatment have also been identified. Cancer often presents as a life‐threatening condition, and understandably patients are anxious about their diagnosis, treatment, prognosis, and disease stage. The SARS‐CoV‐2 outbreak has exacerbated many of these fears, which, along with concerns about developing COVID‐19, has resulted in an increased feeling of insecurity, fragility, and loneliness. Patients mainly reported feelings of anxiety and anger and engaging in non‐functional and, at times, risky behaviors in efforts to manage their distress.

## AUTHOR CONTRIBUTIONS

**Giuseppe Deledda:** Conceptualization (equal); data curation (equal); investigation (equal); methodology (equal); project administration (equal); supervision (equal); writing – original draft (equal); writing – review and editing (equal). **Sara Poli:** Supervision (equal). **Matteo Giansante:** Conceptualization (equal); data curation (equal); investigation (equal). **Eleonora Geccherle:** Supervision (equal). **Giovanna Fantoni:** Supervision (equal). **Incoronata Romaniello:** Conceptualization (equal); supervision (equal). **Farina Gabriella:** Conceptualization (equal); supervision (equal). **Matteo Verzè:** Supervision (equal). **Fabrizio Nicolis:** Supervision (equal). **Stefania Gori:** Supervision (equal); writing – review and editing (equal).

## CONFLICT OF INTEREST STATEMENT

The authors declare no conflict of interest.

## Data Availability

The data that support the findings of this study are available from the corresponding author upon reasonable request.
